# Structural Refinement and Optoelectrical Properties of Nd_2_Ru_2_O_7_ and Gd_2_Ru_2_O_7_ Pyrochlore Oxides for Photovoltaic Applications

**DOI:** 10.3390/ma17112571

**Published:** 2024-05-27

**Authors:** Assohoun Fulgence Kraidy, Abé Simon Yapi, Mimoun El Marssi, Arbelio Penton Madrigal, Yaovi Gagou

**Affiliations:** 1 Laboratoire de Physique de la Matière Condensée, University of Picardie Jules Verne, 33 Rue Saint Leu, CEDEX 01, 80039 Amiens, France; mimoun.elmarssi@u-picardie.fr; 2 UFR Science, Département Science, Structure de la Matière et Technologie, Université Félix Houphouët Boigny, Abidjan BP 258, Côte d’Ivoire; yapiabe@gmx.com; 3 Facultad de Física, IMRE, Universidad de La Habana, San Lázaro y L, Habana CP 10400, Cuba; arbelio@fisica.uh.cu

**Keywords:** pyrochlore oxide, photoanode, conductivity, Raman spectroscopy, structural refinement

## Abstract

High-performance photovoltaic devices require active photoanodes with superior optoelectric properties. In this study, we synthesized neodymium ruthenate, Nd_2_Ru_2_O_7_ (NRO), and gadolinium ruthenate pyrochlore oxides, Gd_2_Ru_2_O_7_ (GRO), via the solid-state reaction technique, showcasing their potential as promising candidates for photoanode absorbers to enhance the efficiency of dye-sensitized solar cells. A structural analysis revealed predominantly cubic symmetry phases for both materials within the *Fd*-*3m* space group, along with residual orthorhombic symmetry phases (Nd_3_RuO_7_ and Gd_3_RuO_7_, respectively) refined in the *Pnma* space group. Raman spectroscopy further confirmed these phases, identifying distinct active modes of vibration in the predominant pyrochlore oxides. Additionally, a scanning electron microscopy (SEM) analysis coupled with energy-dispersive X-ray spectroscopy (EDX) elucidated the morphology and chemical composition of the compounds. The average grain size was determined to be approximately 0.5 µm for GRO and 1 µm for NRO. Electrical characterization via I-V measurements revealed that these pyrochlore oxides exhibit n-type semiconductor behavior, with conductivity estimated at 1.5 (Ohm·cm)^−1^ for GRO and 4.5 (Ohm·cm)^−1^ for NRO. Collectively, these findings position these metallic oxides as promising absorber materials for solar panels.

## 1. Introduction

The urgent global need to mitigate climate change by reducing carbon dioxide emissions has highlighted the critical importance of scalable and sustainable energy sources. Among the various avenues for achieving this goal, the development of high-performance semiconductors for photovoltaic devices stands out as a promising solution. In particular, dye-sensitized solar cells (DSSCs) have emerged as a versatile and cost-effective option for harnessing solar energy [1]. Despite significant progress in DSSC technology, challenges persist in optimizing the performance and stability of these devices. Central to these challenges is the need for robust and efficient photoanode materials that can effectively capture and convert sunlight into electrical charge. Conventionally, nanocrystalline titanium dioxide (TiO_2_) has served as the primary photoanode material in DSSCs, owing to its favorable electronic properties and compatibility with various dye sensitizers [2]. However, the widespread adoption of TiO_2_ photoanodes is hindered by several inherent limitations. For instance, TiO_2_ exhibits a protracted dye absorption time, limiting the overall efficiency of DSSCs. Moreover, its relatively low electron mobility and propensity for charge recombination at the electrolyte interface further diminish device performance. To address these shortcomings, researchers have increasingly turned their attention to alternative semiconductor materials that offer improved optoelectronic properties and enhanced device performance [3]. One promising class of materials that has garnered considerable interest in recent years is pyrochlore oxides. Pyrochlore oxides are complex compounds characterized by a cubic crystal structure with a general formula of A_2_B_2_O_7_, where A and B represent cations occupying distinct crystallographic sites [4]. These materials exhibit a range of desirable properties, including high chemical stability, tunable electronic structure, and favorable optical absorption characteristics in a large optical range. These pyrochlore oxides can exhibit structural phase transitions at low temperatures, and they are controlled by rare earth elements doping [5]. A growing body of research has demonstrated the potential of pyrochlore oxides as viable candidates for photoanode materials in DSSCs. Notably, studies have shown that incorporating pyrochlore phases into photoelectrode architecture can significantly enhance device efficiency and stability. By tailoring the composition and structure of pyrochlore oxides, researchers can achieve precise control over key parameters such as bandgap energy, charge carrier mobility, and charge recombination kinetics [4,6].

In this study, we explore the synthesis and characterization of two pyrochlore oxides, namely neodymium ruthenate (Nd_2_Ru2O_7_) and gadolinium ruthenate (Gd_2_Ru_2_O_7_), as potential photoanode materials for DSSCs. We employ the solid-state reaction technique to prepare these materials and systematically investigate their structural, optical, and electrical properties. Through a combination of advanced analytical techniques, including X-ray diffraction (XRD), Raman spectroscopy, scanning electron microscopy (SEM), and electrical measurements, we elucidate the structural integrity, composition, and electronic behavior of these pyrochlore oxides [7,8]. Our findings offer valuable insights into the suitability of Nd_2_Ru_2_O_7_ and Gd_2_Ru_2_O_7_ as photoanode materials in DSSCs and pave the way for further optimization and integration of these materials into next-generation photovoltaic devices.

## 2. Experimental Techniques

### 2.1. Materials Growth

NRO and GRO powders were synthesized via the solid-state reaction technique [9,10,11,12,13,14,15]. Neodymium (III) oxide (Nd_2_O_3_, purity 99.99%, Sigma-Aldrich, Taufkirchen, Germany), gadolinium (III) oxide (Gd_2_O_3_, purity 99.99%, Sigma-Aldrich), and ruthenium (IV) oxide (RuO_2_, purity 99.99%, Sigma-Aldrich) were employed as starting materials. Stoichiometric quantities of the precursor oxides were weighed and mixed in ethanol before being introduced into a ball milling mortar for two hours to ensure homogeneity and fine grain size. The resulting slurries, following ethanol evaporation, were ground in an agate mortar and then subjected to calcination at a heating rate of 5 °C/min to reach 900 °C with a dwell time of five hours. Pellets were fabricated from the calcined powders and sintered at 1000 °C for 27 h. Subsequently, the sintered pellets were crushed to obtain fine powder particles. For GRO, the heat treatment was conducted in an inert atmosphere (Nitrogen) in two stages: first, for 24 h at 900 °C, followed by 12 h at 1200 °C. The characteristic values of the diameter shrinkages ((initial diameter−final diameter)/initial diameter) were systematically determined to be approximately 11%. The symmetry phases of the pyrochlore oxides were confirmed using an X-ray diffraction (XRD, Bruker, Ettlingen, Germany) analysis with CuKα wavelength radiation (λ = 1.5406 Å) and employing a Bruker D4 apparatus. The morphology of the grains was assessed using scanning electron microscopy (SEM, FEI Quanta 200 FEG, FEI, Hillsboro, OR, USA) with an FEI Quanta 200 instrument equipped with an energy dispersive X-ray (EDX, X-Max 80, Oxford Instruments Co., Abingdon, UK) system, enabling the simultaneous determination of the chemical composition of the material.

Optical measurements were conducted using a JASCO V-670 spectrophotometer, while the current-voltage (I–V) characteristics were obtained using a KEITHLEY SourceMeter 2611A System (Tektronix/Keithley, Cleveland, OH, USA), with the sample connected to a Linkam THS340-type oven (Linkam Scientific Instruments Ltd., Redhill, UK). Raman vibration modes were identified using a RENISHAW inVia Raman spectroscope (Renishaw UK Sales Ltd., Gloucestershire, UK)with a non-polarized laser (λ = 532 nm) [14,15,16,17].

### 2.2. Thermogravimetric and DSC Analysis

**Figure 1:**
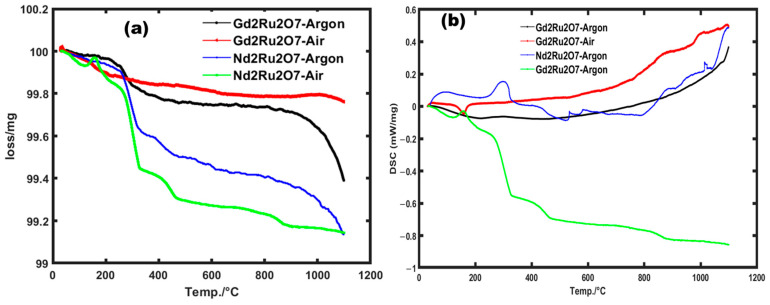
TGA (**a**) and DSC (**b**) curves of Nd_2_Ru_2_O_7_ and Gd_2_Ru_2_O_7._

We conducted a thermal study on NRO and GRO powders to assess their thermal stability by quantifying the mass loss during the formation process of the materials. To investigate their thermal behavior, the samples were subjected to a controlled temperature rise from 30 to 1100 °C at a rate of 5 °C/min. The thermogravimetry analysis (TGA) and dynamical scanning calorimetry (DSC) measurement results are plotted in Figure 1a and Figure 1b, respectively.

We observed a mass loss at 266 °C, resulting in peaks at the same temperature on the DSC curves, and this is attributed to the release of residual oxides produced during the preparation of the powder, which did not participate in the chemical reaction that produced the NRO and GRO oxides. The second mass loss, appearing at 920 °C, can be attributed to the decomposition of reagents (initial oxide precursors) to form the products (new compounds) (Figure 1a). Additionally, the differential scanning calorimetry (DSC) analysis (Figure 1b) revealed an increase in melting and crystallization temperatures of around 1080 °C for GRO in an inert medium. Under air, a transition was observed at 1100 °C for the GRO compound and at 1000 °C for the NRO. These observations indicate the transformation temperature in the process of formation of these materials. Therefore, we hypothesize that 1200 °C could be chosen to attempt the elaboration of single phases in air, starting with the different oxides, with less mass loss (~1% loss) following the reaction equation:

1/2(RE)_2_O_3_ + RuO_2_ → (RE)RuO_3_, with RE = Nd, Gd

However, after several treatments at this temperature, a pyrochlore phase was obtained in the presence of a residual phase, instead of a pure perovskite phase due to experimental growing conditions without pressurization. Surprisingly, this did not significantly affect the electrical and optical performance of these materials. We speculate that the stabilization of the perovskite phase (RE)RuO_3_ could be achieved through a protocol utilizing a reaction chamber under pressure, as reported by A. Sinclair et al. [14].

## 3. Results and Discussions

### 3.1. SEM Analysis of Nd_2_Ru_2_O_7_ and Gd_2_Ru_2_O_7_ Ceramics

**Figure 2:**
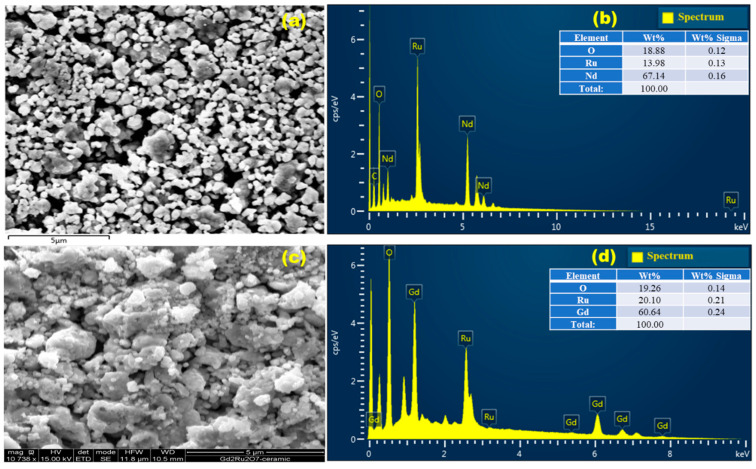
SEM-EDX on ceramics of NRO (**a**,**b**) and GRO (**c**,**d**).

The surface morphology of the elaborated ceramics was examined using scanning electron microscopy (SEM). The particle sizes were estimated to be approximately 1 µm for NRO and 0.5 µm for GRO. SEM images revealed distinct morphological differences between the two materials. For NRO, SEM images showed spherical-shaped grains interspersed randomly between some agglomerated grains (Figure 2c). In contrast, GRO exhibited drastically different morphologies, characterized by strong agglomerations of grains and irregular shapes (Figure 2a).

Using energy-dispersive X-ray spectroscopy (EDX), we confirmed the homogeneously distributed atomic proportions present in the GRO and NRO structures, as shown in Figure 2b,d. The analysis of the studied areas confirmed that the NRO and GRO pyrochlores were composed of the expected chemical elements without impurities [15]. Similar observations have been reported for other pyrochlore-like structures in the literature [16,17].

### 3.2. X-ray Diffraction Study 

A qualitative analysis of both diffraction patterns indicated the presence of two phases. A major cubic phase was identified as (RE)_2_Ru_2_O_7_, space group *Fd*-3*m* (ICSD: 79332) [18,19], and a minor orthorhombic phase as (RE)_3_RuO_7_, space group *Cmcm* (ICSD: 92067), with RE = Gd or Nd. The determination of unit cell parameters as well as the volume fraction of each phase was carried out through Rietveld refinement using the FullProf software (Version 19.04.24) [20,21]. The initial structural models used in the refinement were adopted from reference [22]. The profile adjustment led to rapid convergence with the FullProf software in pattern matching mode. The structure calculation was then conducted using the Rietveld method, which allowed us to refine atomic positions and their occupation rates on the sites to be consistent with the *Fd*-3*m* space group.

#### 3.2.1. Pyrochlore Oxide Nd_2_Ru_2_O_7_

**Figure 3:**
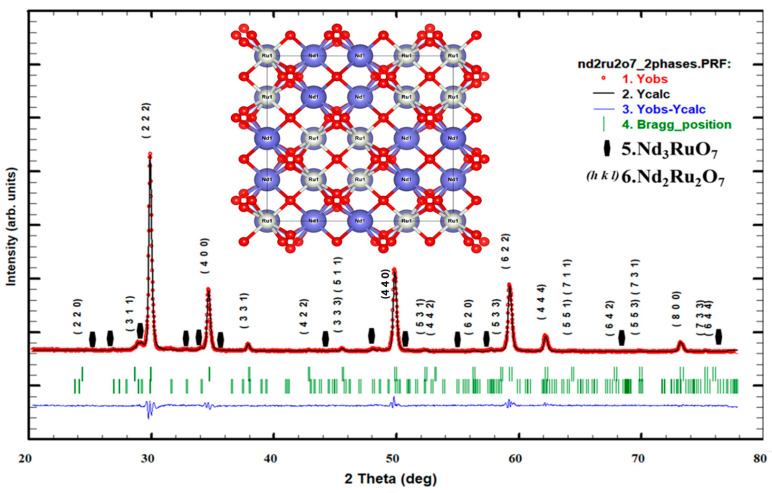
Rietveld refinement plots for Nd_2_Ru_2_O_7_. Solid hexagonal marks indicate the residual phase.

The pyrochlore oxide symmetry of Nd_2_Ru_2_O_7_ was conclusively confirmed from X-ray patterns using a Cu-Kα source [23,24,25,26] in a powder diffraction apparatus. The calculated and simulated X-ray patterns resulting from the Rietveld refinement are shown in Figure 3.

The results show the presence of a minority secondary phase, of which some low-intensity reflections have been highlighted with solid black hexagonal marks. Figure 3 illustrates the Rietveld refinement of the X-ray powder diffraction pattern of NRO. The observed data are denoted with red dots, and the corresponding calculated profiles are represented by the continuous black line. The vertical green lines below the pattern indicate the positions of all permitted Bragg reflections for both phases. At the bottom of the figure, the difference between the observed and the calculated data is plotted in the continuous blue line. Additionally, we present, in the inset of this figure, the geometrical construction of the NRO structure using VESTA software v.3 [27,28].

Table 1 presents the results of the Rietveld refinement of the NRO structure, including atomic positions, lattice parameters, volume, and reliability factors. 

The refinement was performed in the *Fd*-3*m* space group (SG). The structural parameters obtained in this SG are as follows: a = 10.2336 Å, α = β = γ = 90°, and the volume V = 1105.274 Å^3^, with a cubic phase dominance of 93.78%. 

Table 2 shows the results of the Rietveld refinement of the residual phase of the Nd_2_Ru_2_O_7_ structure. The fit was performed in orthorhombic symmetry with the *Cmcm* space group. The parameters obtained are as follows: a = 10.6324 Å, b = 7.3423 Å, c = 7.3763 Å, α = β = γ = 90°, and V = 603.195 Å^3^, that represents a fraction of 6.22%.

#### 3.2.2. Pyrochlore Oxide Gd_2_Ru_2_O_7_

**Figure 4:**
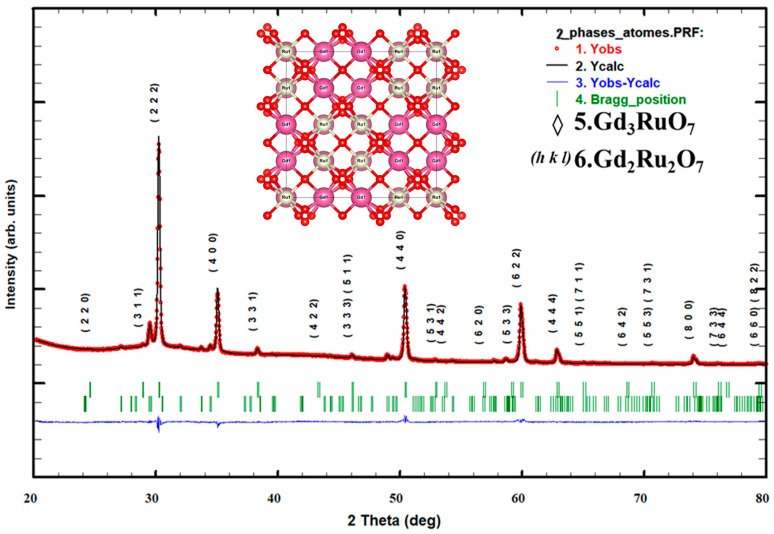
XRD diffraction pattern of Gd_2_Ru_2_O_7_ powder. The opened diamond marks indicate the residual phase.

Figure 4 shows the Rietveld refinement of the X-ray powder diffraction pattern of the GRO samples.

The observed data are denoted with red dots, and the corresponding calculated profiles are represented by the continuous black line. The vertical green marks below the pattern give the positions of all permitted Bragg reflections for both phases. At the bottom of the figure, the difference between the observed and the calculated data is plotted in the blue continuous curve.

In the experimental X-ray pattern, the higher intensities diffraction peaks represent the dominant pyrochlore phase of the Gd_2_Ru_2_O_7_ type [29,30,31,32,33]. Additional diffraction, peaks indicated by the opened diamonds marks, correspond to the Gd_3_RuO_7_ [29,32] pyrochlore and are refined in orthorhombic symmetry with *Cmcm* SG (92067-ICSD). The geometrical representation of the structure that was built using VESTA software is inserted in Figure 4.

Table 3 shows the results of the Rietveld refinement of the Gd_2_Ru_2_O_7_ structure.

The profile adjustment led to rapid convergence with Fullprof software in pattern matching mode. The structure calculation was then conducted using the Rietveld method that permitted us to refine atomic positions, their occupation rates on the sites, and the isotropic thermal agitation factors compatible with *Fd*-3*m* SG. The obtained structure parameters and conventional Rietveld refinement reliability factors are gathered in this table. 

Table 4 shows the results of the structural refinement of the residual phase present in this diagram. The adjustment was conducted in the orthorhombic *Cmcm* SG. 

From this structural analysis, the calculated parameters as well as the conventional Rietveld’s goodness of fit parameters are reported in Table 5 as a comparative investigation.

The behavior of the unit call parameters is consistent with the variation of the ionic radius of the dopants (RE). The results indicate that the dopant also diffuses in the minority phase. For the calculation of the volume fraction of the two phases, it was assumed that both have similar absorption coefficients. The Gd-doped sample significantly favors the formation of the secondary phase.

### 3.3. Raman Study of Nd_2_Ru_2_O_7_ and Gd_2_Ru_2_O_7_ Powder

**Figure 5:**
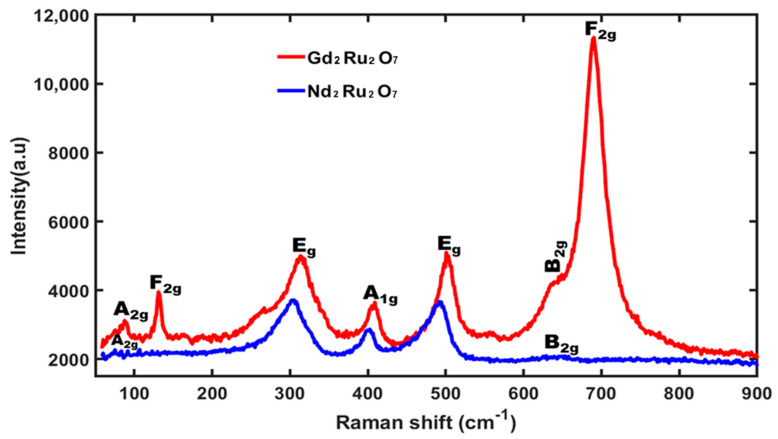
Raman spectrum of Nd_2_Ru_2_O_7_ and Gd_2_Ru_2_O_7_ with λ = 532 nm.

Figure 5 illustrates the Raman spectrum recorded for Nd_2_Ru2O_7_ and Gd_2_Ru_2_O_7_ ceramics. The peak at 75 cm^−1^ is attributed to the A_2g_ vibration mode, previously observed in the Nd_2_O_3_ structure [33] and corresponding to O-Nd-O vibrations. Additionally, the peaks at 87 cm^−1^ and 130 cm^−1^ are attributed to the A_2g_ and F_2g_ modes, identifiable in the Gd_2_O_3_ structure [34] and attributed to O-Gd-O vibrations. 

The peaks at 302 cm^−1^, 402 cm^−1^, 492 cm^−1^, and 645 cm^−1^ can be attributed to the E_g_, A_1g_, and B_2g_ modes previously observed in the RuO_2_ matrix [35,36]. These different vibration modes are visible in the two pyrochlore NRO and GRO oxides symmetries. Specifically, the peak at 645 cm^−1^ represents the vibrational stretching mode of the O-Nd-O-Ru-O bond in the Nd_2_Ru_2_O_7_ structure. Similarly, the peak at 690 cm^−1^ in the Raman spectrum of Gd_2_Ru_2_O_7_ is attributed to the F_2g_ mode, which corresponds to the O-Gd-O-Ru-O vibration. These modes confirm the structural characteristics of Nd_2_Ru_2_O_7_ and Gd_2_Ru_2_O_7_, obtained from the combination of Nd_2_O_3_ and RuO_2_, followed by the vibrational stretching modes that symbolize the presence of defects or oxygen vacancies in these two structures.

The general active modes of the structural symmetry for Nd_2_Ru_2_O_7_ are denoted as G_(Raman NRO)_ = A_1g_ + A_2g_ + B_2g_ + 2E_g_, whereas Gd_2_Ru_2_O_7_ is designated as G_(Raman GRO)_ = A_1g_ + A_2g_ + B_2g_ + 2E_g_ + 2F_2g_. These Raman modes are active in the *Fd*-3*m* space group. The vibrational modes of the majority and minority phases were found to be very close. These modes are often located in the background or are confounded with peaks of predominant phase modes. Vibrational modes A_1g_ and B_2g_ are attributed to the (RE)_3_RuO_7_ minority phases [32] but are also visible in the main phase as observed above.

## 4. Electrical Properties of the Pyrochlore Oxides Nd_2_Ru_2_O_7_ and Gd_2_Ru_2_O_7_

**Figure 6:**
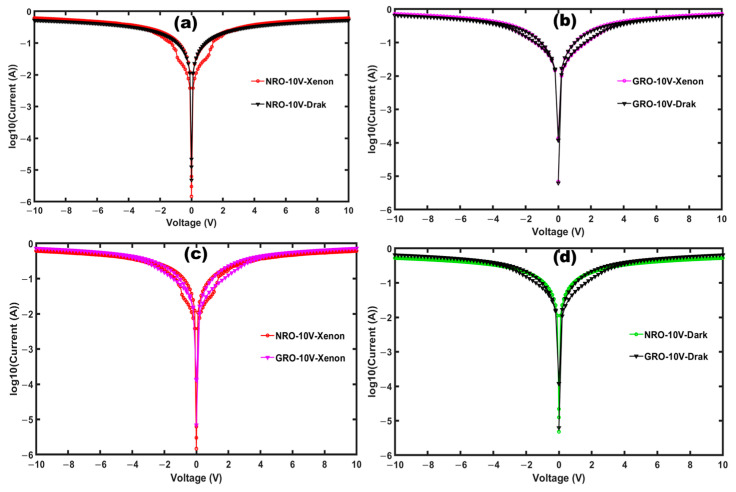
I–V Characteristic curves of NRO in dark and xenon illumination; (**a**) GRO in dark and xenon illumination; (**b**) comparative curves of NRO and GRO currents under xenon illumination (**c**) and in dark (**d**).

Electrical measurements were conducted on NRO and GRO ceramics both in the dark and under illumination from a Xenon lamp (11W-E27: 220-240V, LEDVANCE, Xenon Lamp Ledvance, München, Germany). Platinum electrodes were placed on either side of the ceramic samples. Using a Keithley SourceMeter 2611A System (Keithley 2611 A, Keithley Tektronics Company, Berkshire, UK), characteristic current-voltage (I–V) measurements were performed on these materials. Figure 6 illustrates the I–V curves with a voltage up to 10 V, both in the dark and under Xenon illumination for NRO and GRO. Specifically, Figure 6a,b depict the I–V curves under light and in the dark for NRO and GRO, respectively. NRO, which exhibited simple diode behavior, highlighted an open hysteresis under xenon illumination. Conversely, GRO, which presented a resistance switching effect in the dark, almost enlarged hysteresis under xenon illumination [37,38]. Upon comparing the curves obtained under light and in the dark for both NRO and GRO, it is evident that there is an increase in current density under illumination, indicating the enhanced mobility of charge carriers. This phenomenon can be attributed to the conductivity of the materials, which is facilitated by the movement of charge carriers in the presence of light as charge accumulates at the conduction band. It is noteworthy that the currents observed in both materials are relatively high, which is indicative of n-type semiconductor behavior. This classification of NRO and GRO place them in the category of excitable semimetallic pyrochlore oxides suitable for photovoltaic applications [39,40].

A slight increase in intensity is observed when the material is illuminated with a Xenon lamp compared to measurements made in the dark. The observed opening (hysteresis) in the range −5 to 5 V under illumination is characteristic of a bipolar-type resistive switching mechanism, which can be attributed to several factors, such as the presence of active dipoles in the material, filamentary conduction, or the interaction of defects in the ceramic or oxygen vacancy. The widening of the hysteresis under illumination is typical in these materials, especially for NRO. This phenomenon is clearly visible under illumination and observable in NRO in the dark (without hysteresis), exhibiting simple diode behavior in the dark and then, large opened hysteresis under illumination. The hysteresis region is localized clearly in the range between (−3V and 3V) before reaching a saturation effect ranging in ±(5 to 10 V). The replacement of neodymium (Nd) by gadolinium (Gd) on the same pyrochlore site A increases the current intensity through the material, although this is at room temperature.

**Figure 7:**
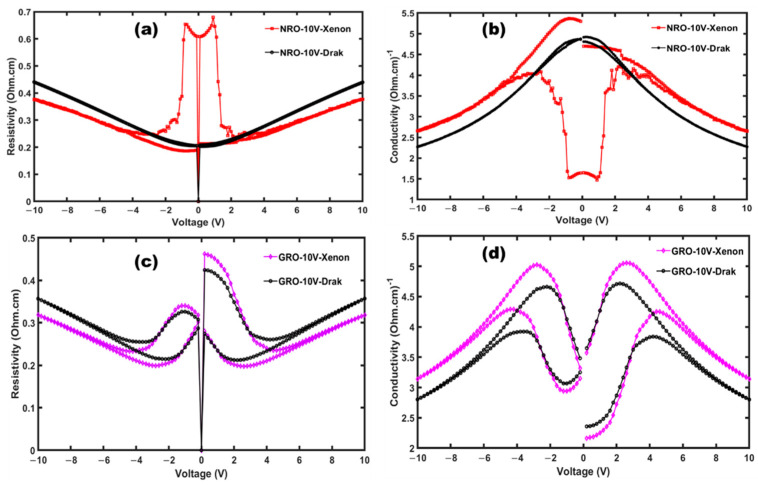
Resistivity (**a**,**c**) and conductivity (**b**,**d**) under xenon illumination and dark for NRO and GRO, respectively.

Figure 7 illustrates the resistivity (Figure 7a,c) and conductivity (Figure 7b,d) under xenon and dark conditions for NRO and GRO. In both cases, it observable that under xenon illumination, resistivity is lower than it is in the dark, which means conductivity is higher for both materials under xenon illumination. Theses result confirm the photoactivity behavior in both compounds.

**Figure 8:**
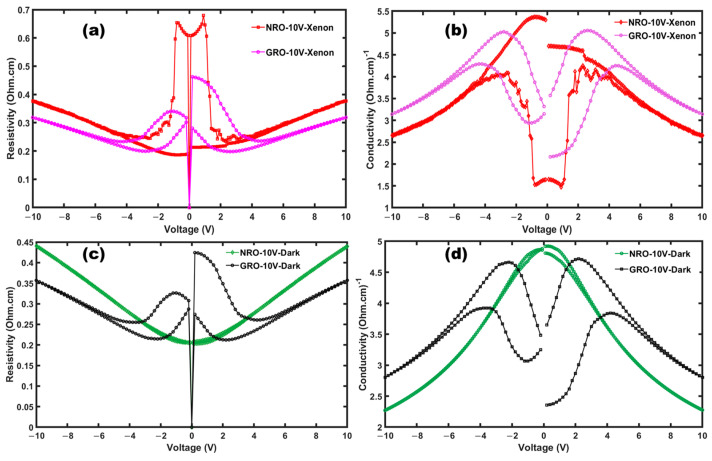
Resistivity and conductivity under xenon (**a**,**b**); resistivity and conductivity in dark (**c**,**d**).

In Figure 8, we can now compare the resistivity and conductivity of NRO and GRO under illumination (Figure 8a and Figure 8b, respectively) and in the dark (Figure 8c and Figure 8d, respectively). It is observed that NRO is more resistive than GRO, and conversely, its conductivity is lower (1.5 (Ohm·cm)^−1^) compared to Gd_2_Ru_2_O_7_ (4.5 (Ohm·cm)^−1^). Furthermore, we observe the stable activity of GRO near the hysteresis region compared to NRO, which exhibits chaotic variation under illumination in the hysteresis region. This can be attributed to grain size effects. Indeed, as shown in the SEM images, NRO presents a less compact microstructure (Figure 2a).

Haouzi et al. [41] demonstrated that the resistivity of Nd_2_Ru_2_O_7_ drops by a factor of 10^3^ when Nd is replaced by Cu in the Nd_2_Ru_2_O_7_ matrix, resulting in metallic-type conductivity. Theoretical calculations within the framework of the local density approximation, carried out in the work of Castro et al. [42], reveal that the compound GRO is a semiconductor with an activation energy (*E_a_*) of 0.089 eV. The high conductivity observed in these pyrochlore oxides can facilitate charge transfer for photovoltaic and photocatalytic applications [43]. Figure 6 illustrates a bipolar resistive switching mechanism, which is less observable in NRO in the dark. Around 0 V, there is an instability in the system for both materials, which is more significant in NRO under xenon illumination. The resistive switching behavior, combined with semiconductor behavior, is interesting for photoanode activity, since it will contribute to high open circuit voltage and also be favorable for electrochemical battery applications.

## 5. Optical Properties of GRO and NRO

**Figure 9:**
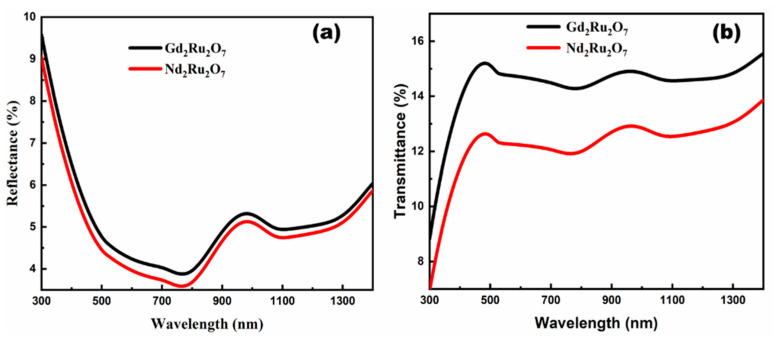
Reflectance (**a**) and transmittance (**b**) of GRO and NRO powders.

The transmittance and reflectance measurements of GRO and NRO powders were conducted using a JASCO V-670 spectrophotometer. Figure 9 illustrates the percentage reflectance of both materials (Figure 9a) and the transmittance (Figure 9b) of both GRO and NRO powders.

**Figure 10:**
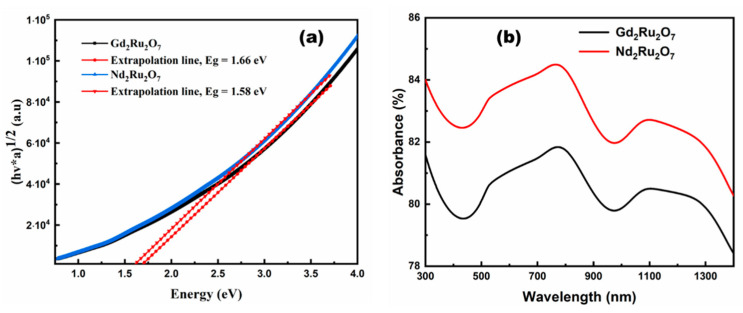
Tauc’s diagram (**a**) and absorbance (**b**) for GRO and NRO.

The spectra show that both samples transmit weakly over the visible to near-infrared range (350–1000 nm). The quantified reflectance value of GRO is 1.04% higher than that of NRO, indicating that NRO reflects less light in the absorption range than GRO. The optical energy gap (*E_g_*) of GRO and NRO powders was evaluated using Tauc’s law [44]:

(1) $$\alpha h\nu =A{\left(h\nu -E_{g}\right)}^{a}$$

where *A* is a constant, and *a* is the factor which indicates the type of electronic transition. In the case of the direct allowed transition, *a *= 1/2, and in the indirect allowed transition, *a *= 2. The energy gap in the samples, which are due to direct electronic transitions, and the direct permitted transition allowed the best fits at *a* = 1/2. These plots were used to determine (*E_g_*) values for GRO and NRO samples, which are 1.66 eV and 1.58 eV, respectively (see Figure 10a,b). Figure 10b shows the absorbance of the GRO and NRO powders. These samples all absorb in the near-infrared, confirming their importance for photovoltaic applications. GRO absorbs less than 0.96 units compared with NRO, but both samples absorb in the same wavelength range (350–1000 nm).

## 6. Conclusions

The development of new pyrochlore oxide materials through the solid-solid synthesis method has resulted in the dominant (RE)_2_Ru_2_O_7_ structure in the absence of pressured equipment. In this study, we employed a cost-effective elaboration and heat treatment process while optimizing the overall procedure. A structural analysis based on X-rays, SEM/EDX, and Raman spectroscopy enabled us to determine the symmetries of pyrochlore oxides, identify atomic positions, and reveal residual phases. Electrical characterization indicated that both materials exhibit n-type semiconductor behavior, with a conductivity estimated at 1.5 (Ohm·cm)^−1^ for GRO and 4.5 (Ohm·cm)^−1^ for NRO. Optical measurements confirmed that both samples absorb in the same wavelength range (350–1000 nm), with bandgap energy E_g_ values calculated for GRO and NRO at 1.66 eV and 1.58 eV, respectively. The presence of oxygen vacancies and defects was a contributing factor to the high conductivity and resistance switching effects observed in these materials. Based on the obtained results, it is concluded that both GRO and NRO materials have potential to be used as photoanodes in photovoltaic devices.

## Figures and Tables

**Table 1:** Atomic positions for Nd_2_Ru_2_O_7_ in *Fd*-3*m* Space Group.

*Atoms*	*x*	*y*	*z*	*Occ*	*Mult.*
*Nd1*	0.5	0.5	0.5	0.08	16
*Ru1*	0.0	0.0	0.0	0.08	16
*O1*	0.332	0.125	0.125	0.25	48
*O2*	0.375	0.375	0.375	0.041	8
*Lattice parameters*	a = 10.2336 Å α = β = γ = 90°				
*Reliability factors*	*R_P_* = 13.6	*R_WP_* = 11.6	*R_F_* = 2.64	χ^2^ = 1.17	
*Unit cell* *Volume V (Å^3^)*	V = 1105.2682 Å^3^				
*Nb Refined* *parameters*	11				
*Ru-O1*	1.969 (1) Å				
*Ru-O2*	4.286 (4) Å				

**Table 2:** Atomic residual positions for Nd_2_Ru_2_O_7._

*Atoms*	*x*	*y*	*z*	*Occ*	*Mult.*
*Nd1*	0.0	0.0	0.0	0.25	4
*Nd2*	0.224(1)	0.302(4)	0.2500	0.50	8
*Ru1*	0.0	0.05	0.0	0.25	4
*O1*	0.128(2)	0.316(9)	0.9590	1.00	16
*O2*	0.133(5)	0.271(0)	0.2500	0.50	8
*O3*	0.000	0.414(9)	0.2500	0.25	4
*Lattice parameters*	a = 10.8989 Å b = 7.3894 Å c = 7.4899 Å				
*Reliability factors*	*R_P_* = 13.6	*R_WP_* = 11.6	*R_F_* = 2.64	χ^2^ = 1.18	
*Unit cell* *Volume V (Å^3^)*	V = 603.204(8) Å^3^				
*Nb Refined* *parameters*	11				
*Ru1-O1*	1.969(0) Å				
*Ru1-O3*	1.975(2) Å				

**Table 3:** Atomic positions for the Gd_2_Ru_2_O_7_ compound.

*Atoms*	*x*	*y*	*z*	*Occ*	*Mult*
*Gd1*	0.5	0.5	0.5	0.083	16
*Ru1*	0.0	0.0	0.0	*0.083*	16
*O1*	0.332	0.125	0.125	*0.250*	48
*O2*	0.375	0.375	0.375	*0.041*	8
*Lattice parameters*	a = b = c = 10.233638 Å, α = β = γ = 90°				
*Reliability factors*	*R_P_* = 12.4	*R_WP_* =7.25	*R_F_* = 2.64	χ^2^ = 7.55	
*Unit cell* *Volume V (Å^3^)*	V = 1071.742(3) Å^3^				
*Nb Refined* *parameters*	12				
*Ru-O1*	1.994(2) Å				

**Table 4:** Atomic residual positions.

*Atoms*	*x*	*y*	*z*	*Occ*	*Mult*
*Gd1*	0.0	0.0	0.0	*0.25*	4
*Gd2*	0.2241	0.3024	0.2500	*0.5*	8
*Ru1*	0.0	0.05	0.0	*0.25*	4
*O1*	0.1282	0.316(9)	0.95900	*1*	16
*O2*	0.13350	0.2710	0.2500	0.5	8
*O3*	0.000	0.4149	0.2500	0.25	4
*Lattice parameters*	a = 10.6323 Å, b = 7.3423 Å, c = 7.3763 Å, α = β = γ = 90°				
*Reliability factors*	*R_P_* = 12.4	*R_WP_* = 7.25	*R_F_* = 2.64	χ^2^ = 7.55	
*Unit cell* *Volume V (Å^3^)*	V = 1071.7423 Å^3^				
*Nb Refined* *parameters*	12				
*Ru-O1*	1.994(2) Å				
*Ru-O2*	4.242(6) Å				

**Table 5:** Unit cell parameters, volume fractions, and goodness of fit parameters.

Identified Phases	Unit Cell Parameters (Cubic Phase)/Å	Unit Cell Parameters (Orth. Phase)/Å	Vol. Fraction	Rp/Rwp Values
Gd_2_Ru_2_O_7_ (cub.) Gd_3_RuO_7_ (orth.)	a = 10.233(5)	a = 10.631(9) b = 7.342(7) c = 7.375(7)	Cub.: (85.49 ± 0.22) % Orth.: (14.51 ± 0.14) %	11.0/6.1
Nd_2_Ru_2_O_7_ (cub.) Nd_3_RuO_7_ (orth.)	a = 10.339(2)	a = 10.898(5) b = 7.389(6) c =7.489(8)	Cub.: (93.78 ± 0.38) % Orth.: (6.22 ± 0.14) %	13.6/11.6

## Data Availability

The original contributions presented in the study are included in the article, further inquiries can be directed to the corresponding authors.
